# Low-Dose Cyclophosphamide Induces Nerve Injury and Functional Overactivity in the Urinary Bladder of Rats

**DOI:** 10.3389/fnins.2021.715492

**Published:** 2021-10-01

**Authors:** Rui Wang, Ming Hong, Jingyi Huang, Na Zhou, Yao Zhang, Siyuan Xu, Jiaye Liu, Junjie Yuan, Lusiqi Zhang, Linyuan Huang, Ping Huang, Bo Tan, Hong-ying Cao

**Affiliations:** ^1^School of Pharmaceutical Sciences, Guangzhou University of Chinese Medicine, Guangzhou, China; ^2^School of Basic Medical Sciences, Guangzhou University of Chinese Medicine, Guangzhou, China; ^3^Dongguan & Guangzhou University of Chinese Medicine Cooperative Academy of Mathematical Engineering for Chinese Medicine, Guangzhou University of Chinese Medicine, Guangzhou, China

**Keywords:** urinary bladder, cyclophosphamide, nerve injury, overactive bladder, NLRP6 inflammasome, neuron

## Abstract

**Aim:** This research aimed to investigate the neurotoxicity of low-dose cyclophosphamide (CYP) on the urinary bladder of rats by *in vivo* and *in vitro* studies.

**Methods:** To establish CYP-induced cystitis rat model, rats were treated with three intraperitoneal injections of CYP (25 mg/kg) in a week. During treatment, the up-down method was used to assess the mechanical withdrawal threshold. On day 8, urodynamic test and bladder smooth muscle contractility study, including the contraction of bladder strips to electrical field stimulation (EFS, 2–64 Hz), carbachol (CCh, 10^–8^–10^–5^ M) and KCl (120 mM), were performed to evaluate the function of bladder function. Body weight and bladder weight were also recorded. Morphometric analysis using an optical microscope and transmission electron microscope was performed to observe the changes of microstructure and submicrostructure of the bladder. The major pelvic neurons were isolated and treated with acrolein (the main CYP metabolite) to assess apoptosis *in vitro*. RT-PCR assays were used to quantify the mRNA expression levels of *Nlrp6, Asc, Casp11* and *Casp1* in bladder tissues and primary neurons.

**Results:** After CYP injections, the body weights decreased, but the bladder weights increased in the model group. The mechanical withdrawal threshold of the cystitis model remained at a low level. The morphometric analysis suggested bladder inflammation and neuroinflammation in the bladder of the cystitis rat model. Urodynamic test revealed that, the amplitude, the pressure baseline, the peak pressure and pressure threshold of model rats significantly increased after CYP treatment. The muscle strips of model rats exhibited significantly higher contractility caused by EFS and CCh than the controls. Apoptotic cells appeared at the highest concentration group (100 μM acrolein) after 6 h of acrolein incubation in apoptosis assay of primary neurons. The mRNA expression levels of *Nlrp6* and *Casp11* were significantly increased in the cystitis rat model and in the acrolein-treated neurons.

**Conclusions:** Low-dose CYP treatment was confirmed to induce nerve injury, which leading to bladder pain and overactive bladder in female rats, and the up-regulation of *Nlrp6* and *Casp11* may contribute to these pathological changes.

## Introduction

Cyclophosphamide (CYP) is on the World Health Organization’s List of Essential Medicines and used as chemotherapy or to suppress the immune system (Dugani et al., 2018). When patients take CYP, acrolein, the main metabolite of CYP, accumulates in the bladder during urine storage. Acrolein, which is a highly reactive unsaturated aldehyde, prolonged contact with the bladder wall would generates sterile inflammation in bladder (Moghe et al., 2015). Therefore, one of the most common adverse effects of CYP is urotoxicity, which can range from chronic bladder pain and bladder hyperactivity to bladder fibrosis and severe hemorrhage. These symptoms seriously affect patients’ quality of life and even threaten their lives. As such, these local urological effects are one of major limiting factors in the clinical use of CYP (Fukuoka et al., 1991; Emadi et al., 2009).

Bladder function is mediated mainly by nerve signals from the brain to the bladder nerves, which imbedded within the bladder wall. With the lack of enzymes that metabolize reactive aldehydes, neurons are particularly vulnerable to oxidative stress and neuroinflammation under CYP-treatment (Hamann and Shi, 2009; Iqubal et al., 2019). In fact, treatment with CYP did significantly enhanced total bladder afferent activity to distension, and did not affect spontaneous muscle activity, or the total amount of ATP or ACh released from either the lumen or the serosa of the mouse bladder in a previous study (Mills et al., 2020). Since neurogenic overactive bladder has been reported in CYP-treated rodents, and visceral pain is a common side effect in the clinic treatment of CYP, we suspect that nerve injury has happened in the development of cystitis caused by the CYP administration (Juszczak et al., 2009; Wang et al., 2017; Chen et al., 2019). Neural impairment in the bladder would lead to vesical denervation which can be hard to reverse by medical or surgical intervention. However, studies on the neurotoxicity of CYP usually focused on the central lesion, and overlooked the damage on the bladder nerves (Rzeski et al., 2004; Iqubal et al., 2019).

Recently, Robin found that denervation induced by NLRP3 inflammasome, during bladder outlet obstruction, provokes bladder overactivity in rats (Lutolf et al., 2018). Over the past decade, the inflammasomes were proved to be important mediators of bladder pathology (Purves and Hughes, 2016). NLRP6, a non-canonical inflammasome components, has been predicted to be a useful therapeutic target in CYP-induced cystitis (Inouye et al., 2018). This inflammasome is composed of activated NLRP6 combined with Caspase 11, Caspase 1 and the adaptor ASC, which promoted caspase-1 activation and IL-1β/IL-18 maturation (Hara et al., 2018). It has been reported that murine enteric infections resulted in a rapid and persistent loss of intrinsic enteric-associated neurons via NLRP6 inflammasome (Matheis et al., 2020). We speculate that NLRP6 inflammasome plays a significant role in inflammation-induced neuronal damage.

Therefore, we designed experiments to explore the neurotoxicity of CYP treatment in the bladder of rats by observing the functional and morphological changes of the bladder, and the alternations of NLRP6 inflammasome expression levels in a low-dose CYP-induced cystitis model.

## Materials and Methods

### Reagents and Materials

CYP was purchased from Sigma-Aldrich (United States). Acrolein was purchased from Aladdin (China). Carbachol was obtained from Shandong Bausch & Lomb Freda Phar. Co., Ltd. (China). FastQuant RT Kit (with gDNAse) and Talent qPCR PreMix (SYBR Green) were purchased from TIANGEN Biotech (Beijing) Co., Ltd. (China). TRIzol reagent was purchased from Thermo Fisher Scientific (United States). Von-Frey filaments was obtained from North Coast (United States). Collagenase type II was purchased from Sigma (United States). Bovine serum albumin, neurobasal medium, B27 supplement, fetal bovine serum, antibiotic/antimycotic and L-glutamine were purchased from Gibico (United States), glia-derived neurotrophic factor was purchased from Peprotech (United States). Meilun One Step TUNEL Apoptosis Assay Kit (FITC) was purchased from Dalian Meilun Biotechnology Co., Ltd. (China). Antibody β-III-tubulin was purchased from Affinity Biosciences (China).

### Animal Model

All experimental protocols and animal procedures complied with institutional guidelines and were approved by the ethical principle of the National Research Council. Female Sprague-Dawley rats (180–200 g) were randomly assigned to two groups namely, CYP (n = 14) and control (*n* = 14) groups, and housed in the Experimental Animal Center of Guangzhou University of Chinese Medicine under room temperature and 12 h/12 h light–dark condition. To establish the cystitis rat model, the CYP group was treated with intraperitoneal injection of CYP (25 mg/kg) on days 1, 4, and 7, whereas the control group was injected with saline following a previous study (Chen et al., 2019).

### Assessment of Pain Response

The up-down method was used on both groups to assess pain response, resulting from CYP injections. The mechanical withdrawal threshold of the lower abdomen was measured using a series of von-Frey filaments (range in force at 0.6, 1, 1.4, 2, 4, 6, 8, 10, 15, and 26 g) after 4 h after the injections. “Licking behavior” was considered a positive response.

### Urodynamic Test *in vivo*

Urodynamic test was performed on both groups on day 8. The animals were anesthetized by intraperitoneal injection of urethane (1.2 g/kg). A tubing with an outer diameter of 1 cm was inserted into the bladder through the urethra and connected to a three-way valve. As for the other two ends, the valve was linked to a syringe pump and a pressure transducer. The bladder was filled with 0.9% saline at a rate of 0.08 mL/min. The bladder was emptied and then refilled after an adaptive phase of 10 min. During three urinations, the peak pressure (PP), the amplitude (AMP, the difference between PP and the basal pressure during each contraction period), pressure baseline (PB, the pressure immediately after the reflex contraction), pressure threshold (PT, the immediate pressure just before the reflex contraction), and intercontraction interval (ICI, the average time between contractions of reflex bladder contractions) were recorded.

### Assessment of Bladder Strips Contractility *in vitro*

Krebs–Henseleit solution (NaCl, 118 mM; KCl, 4.75 mM; MgSO_4_, 1.18 mM; NaHCO_3_, 24.8 mM; KH_2_PO_3_, 1.18 mM; CaCl_2_, 2.5 mM; and C_6_H_12_O_6_⋅H_2_O, 10 mM; pH, 7.4) was prepared. The rats were euthanized by carbon dioxide euthanasia, and the urinary bladder was quickly removed and transferred to precooled Krebs–Henseleit solution. Full-thickness longitudinal bladder tissue strips (1 mm × 5 mm) were obtained and suspended in an organ bath filled with Krebs–Henseleit solution at 37°C and bubbled with carbogen (95% oxygen, 5% CO_2_). In the whole assessment process, DSM strips were connected to the force signal transducer to record tensile strength change. The resting tension was loaded at 1 g and the strips were equilibrated for 60 min. The spontaneous contractions (Figure 3K) and contractions of bladder strips to electrical field stimulation (EFS, 2, 4, 8, 16, 32, and 64 Hz; 40 V; and 0.5 ms pulse duration for 10 s), carbachol (CCh, 10^–8^–10^–5^ M) and KCl (120 mM) was recorded successively after every equilibration. To obtain a cumulative concentration–response curve to carbachol, there is a 1-minute interval before the next concentration of carbachol (10^–8^, 3^∗^10^–8^, 10^–7^ and 3^∗^10^–7^ M carbachol), and then a 0.5-minute interval before the next concentration of carbachol (10^–6^, 3^∗^10^–6^ and 10^–5^ M carbachol).

The carbachol curves were cumulative, and the base value used in carbachol analysis was the baseline immediately before the beginning of the curve. Moreover, pEC50 values were calculated according to the formula: pEC50 = −log^10^(EC50). In the end, the weight and length of each DSM strips were recorded, and the formula: [(peak value – base value) (g) × Length (cm) × 1.06 (mg/mm^3^) × 0.0098 (N/g)]/weight (g) was used to normalize the amplitude of contractility.

### Histological Test

The rats were euthanized by carbon dioxide euthanasia, and the urinary bladder was harvested and weighed. After fixation in 4% paraformaldehyde solution for approximately 24 h at room temperature, the bladders were conventionally dehydrated and embedded in paraffin. Then, the tissues were transected along the transverse sections (6 μm) and stained with hematoxylin and eosin (H&E) or Masson’s trichrome. The bladder wall thickness (BWT) was measured on the basis of Masson’s trichrome images. The smooth-muscle-to-collagen ratio was calculated as “red” smooth muscle to “blue” collagen in Masson’s trichrome images, based on the open-resource image software ImageJ and an associated color deconvolution plugin. All images were processed and analyzed with image analysis software (Image Pro 6.0).

### Morphometric Analysis by Electron Microscopy

After the rats were sacrificed, the urinary bladders were fixed with 2.5% glutaraldehyde at 4°C for 4 h. Then, the samples were post-fixed with 1% osmic acid in 0.1 M phosphate buffer at 20°C for 2 h. followed by dehydration and embedding in Epon812 medium. Ultrathin sections (70 nm) were mounted to uranyl acetate and counterstained with lead citrate for electron microscopy. The treated samples were investigated by using a transmission electron microscope (Hitachi).

### Neuronal Isolation, Culture and Apoptosis Assay

Major pelvic ganglia were dissected from naive rats and dissociated with collagenase type II (2 mg/ml) and bovine serum albumin (0.6 mg/ml) for 30 min. They were then cultured on poly-D-lysine and laminin-coated coverslips. The cell medium comprises neurobasal medium, B27 supplement, fetal bovine serum, glia-derived neurotrophic factor, antibiotic/antimycotic and L-glutamine. After 72 h, pelvic neurons were treated with acrolein (the CYP metabolite, 0, 25, 50, and 100 μM) for 6 h to confirm a stimulation concentration of acrolein. After immunofluorescent staining of neuron-specific class III beta-tubulin to identify neurons, apoptosis assay was conducted using the Meilun One Step TUNEL Apoptosis Assay Kit (FITC). Images were visualized with a laser scanning confocal microscope and captured by ZEN software.

### Real-Time RT-PCR

The total ribonucleic acid (RNA) of treated bladder tissues and pelvic neurons (acrolein treatment, 0, 100 μM) was extracted using the TRIzol reagent. The RNA samples were evaluated by ultraviolet absorption at 260 and 280 nm. The A260/A280 was used to check the purity, and the concentration of RNA was confirmed using the A260 values. Then, the RNA samples were reverse transcribed into cDNA using a PrimeScript RT Reagent Kit with gDNA eraser. Synthetic oligonucleotide primers were designed to amplify cDNA for the genes encoding the *Gapdh, Nlrp6, Asc, Casp11* and *Casp1*. Primer pairs were listed in Table 1. A real-time RT-PCR assay was performed using Talent qPCR PreMix (SYBR Green) in the ABI Prism 7500 system (Applied Biosystems, United States). The results were expressed as mRNA levels of each gene studied, and each result was normalized according to *Gapdh* expression.

**TABLE 1:** Primer sequences of *Nlrp6*, *Asc*, *Casp11*, *Casp1* and *Gapdh*.

**Gene**	**Primers (5′–3′)**
*Nlrp6 - F*	CCGCATCGTCTACTGTTCATCCTG
*Nlrp6 - R*	CGATGCTCACCGAACTCTCACG
*Asc - F*	TGTGCTTAGAGACATGGGCATACAG
*Asc - R*	AACGACCTACGAGACATACCG
*Casp11 - F*	CTTCACAGTGCGAAAGAACTG
*Casp11- R*	GTTTTACGAAGAGGTCTGTAAAGAATTACACCTGAG
*Casp1 - F*	CTGGAGCTTCAGTCAGGTCC
*Casp1- R*	CTTGAGGGAACCACTCGGTC
*Gapdh - F*	GACATGCCGCCTGGAGAAAC
*Gapdh - R*	TGATTTCCCGTAGGACCCGA

### Statistical Analysis

All data were expressed as mean ± standard error of the mean (SEM). SPSS Statistics 20.0 was used for statistical analysis. In this study, the independent-samples *T* test was performed, and *P* < 0.05 considered as statistically significant.

## Results

### Pain Response Resulted From Cyclophosphamide Treatment

**FIGURE 1:**
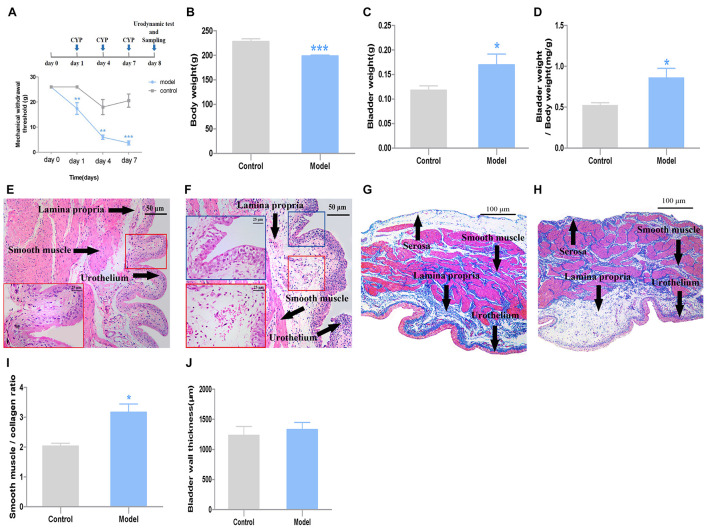
General characteristics and pathological changes of the two groups (*n* = 7). **(A)** Mechanical threshold; **(B)** body weight; **(C)** bladder weight; **(D)** bladder weight index, which was calculated as bladder weight to body weight; **(E)** H&E images of bladder tissues from control rats; **(F)** H&E images of bladder tissues from CYP-induced cystitis model rats, slight epithelial damage, mild bladder mucosa edema and hemorrhage could be observed in the bladder; **(G)** Masson’ s trichrome staining of bladders from control rats; **(H)** Masson’ s trichrome staining of bladders from CYP-induced cystitis model rats; **(I)** smooth-muscle-to-collagen ratio determined by the Masson’s trichrome images, which was calculated as “red” smooth muscle to “blue” collagen; **(J)** BWT measured from Masson’ s trichrome images. Scale bar: 50 and 25 μm. Data represent the means ± SEM (model vs. control group, ***P < 0.001, **P < 0.01 or *P < 0.05).

Following CYP treatment, the mechanical threshold of the model group gradually decreased from day 1 to day 4 compared with that of the control group (*P* < 0.01, Cohen’s *d* > 0.8) (Figure 1A). Then, a significant reduction in the mechanical threshold of the model group was observed after the third CYP injection on day 7 (*P* < 0.001, Cohen’s *d* > 0.8) (Figure 1A).

### General Characteristics and Pathological Changes of the Cystitis Rat Model

Compared with the rats in the control group, the body weight of the model group decreased after three injections (*P* < 0.001, Cohen’s *d* > 0.8) (Figure 1B). Meanwhile, the bladder weight of the model group was greater than those of the control group (*P* < 0.05, Cohen’s *d* > 0.8) (Figure 1C). Accordingly, the bladder mass index (bladder wight to body wight) of model group increased compared with the control group (*P* < 0.01, Cohen’s *d* > 0.8) (Figure 1D).

The histological test results from H&E images showed, slight epithelial damage, mild bladder mucosa edema and hemorrhage were observed in the bladder of the cystitis rat model (Figures 1E,F). The smooth-muscle-to-collagen ratio was increased in the bladder of the model group (*P* < 0.01, Cohen’s *d* > 0.8) (Figure 1I). However, the BWT showed slight difference between the two groups (*P* > 0.05, Cohen’s *d* > 0.2) (Figure 1J) measured from Masson’ s trichrome images (Figures 1G,H).

**FIGURE 2:**
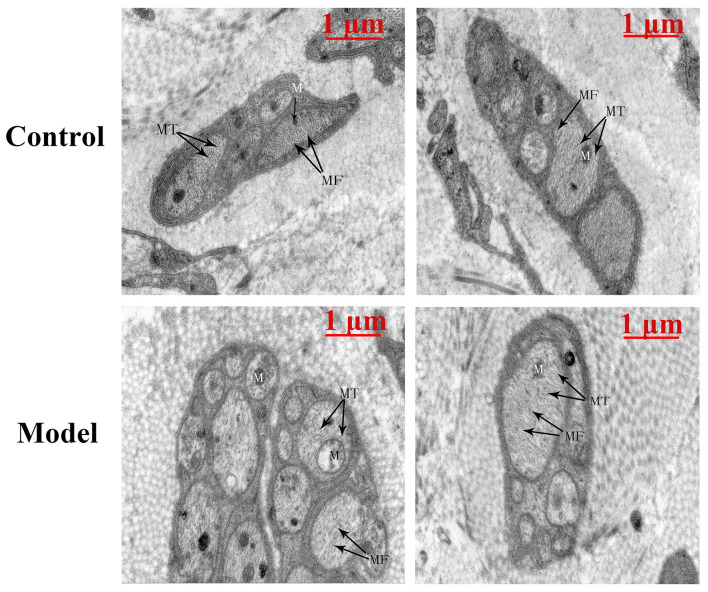
The TEM-image analysis of bladders from the two groups (*n* = 1). The bladder of the cystitis rat model showed, unmyelinated nerve cells exhibited slightly edema, accompanied with swelling organelles. Moreover, neural microfilaments (MF) and microtubules (MT) were arranged disorderly and sparsely in small local areas of the nerve cells. Meanwhile, a few swollen mitochondria (M) could be observed. The matrix in the membrane was dissolved, and the crest was reduced and disappeared. Scale bar: 1 μm.

Under electron microscopy, the bladder of the cystitis rat model showed, unmyelinated nerve cells exhibited slightly edema, accompanied with swelling organelles. Moreover, neural filaments and microtubules were arranged disorderly and sparsely in small local areas of the nerve cells. Meanwhile, a few swollen mitochondria could be observed. The matrix in the membrane was dissolved, and the crest was reduced and disappeared (Figure 2).

### Urodynamic Tests

**FIGURE 3:**
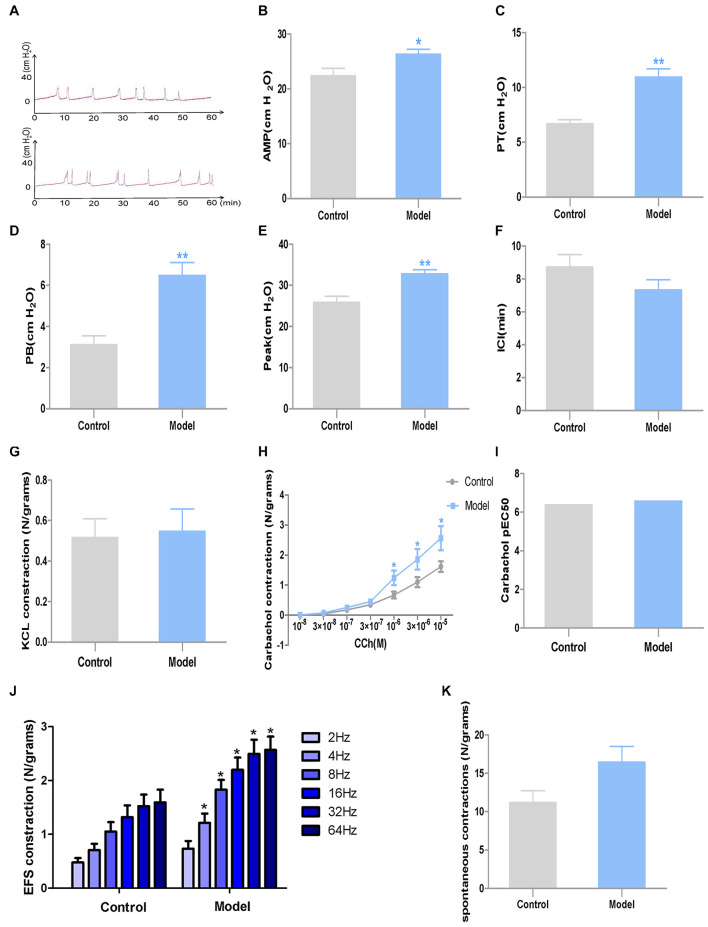
Urodynamic test and DSM strips contractility study results in the two groups (*n* = 7). **(A)** Representative urodynamic test recording from the two groups of mice; **(B)** AMP; **(C)** PT; **(D)** PB; **(E)** peak; **(F)** ICI; **(G)** DSM strip contractions induced by KCl (120 mmol/L); **(H)** DSM strip contractions induced by CCh (10^–8^–10^–5^ M); **(I)** Potency (pEC50) obtained from concentration–response curves to carbachol. **(J)** DSM strip contractions induced by EFS (2–64 Hz); **(K)** Amplitudes of spontaneous contractions. Data represent the means ± SEM (model vs. control group, *P < 0.05 or **P < 0.01).

Urodynamic test revealed that compared with the control group, the PB, PP and PT of model rats all significantly increased after CYP treatment (*P* < 0.01, Cohen’s *d* > 0.8). In the meantime, the AMP of model rats exhibited a moderate growth (*P* < 0.05, Cohen’s *d* > 0.5). However, the ICI of model rats showed slight difference between the two groups (*P* > 0.05, Cohen’s *d* > 0.2) (Figures 3A–F).

### Bladder Strips Contractility Studies

The muscle strips of model rats exhibited significantly higher contractility caused by EFS (4–64 Hz) and CCh (10^–6^–10^–5^ M) than those of control rats (*P* < 0.05, Cohen’s *d* > 0.8). However, there is no substantial differences were observed between the two groups when responding to KCl (120 mM) (*P* > 0.05, Cohen’s *d* > 0.5), lower frequency of EFS (*P* > 0.05, Cohen’s *d* > 0.5) or lower concentration of CCh (*P* > 0.05, Cohen’s *d* > 0.2), while potency (pEC50) to carbachol and spontaneous contractions (Figure 3K) were steady. And spontaneous contractions showed no difference between the two groups (*P* > 0.05, Cohen’s *d* < 0.2) (Figures 3G–J).

### Apoptosis Assay of Primary Neurons

**FIGURE 4:**
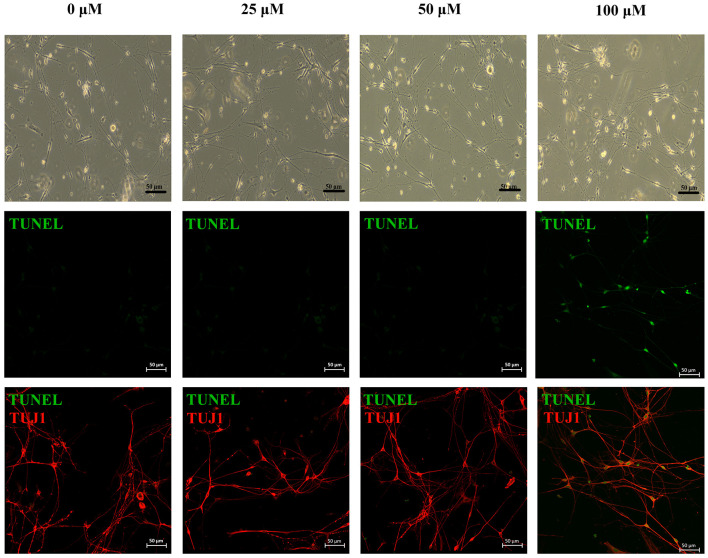
Phase-contrast images and immunofluorescence images of primary neurons treated under different concentrations of acrolein treatment. Apoptotic cells were stained for TUNEL (green, apoptotic marker), after immunofluorescent staining of TUJ1 (red; neuron-specific class III beta-tubulin) to identify neurons. Scale bar: 50 μm.

As shown in the fluorescent images, apoptotic cells appeared at the highest concentration group (100 μM acrolein) after 6 h of acrolein incubation. Meanwhile, those cells were identified as neurons by class III beta-tubulin staining (Figure 4).

### Real-Time RT-PCR

**FIGURE 5:**
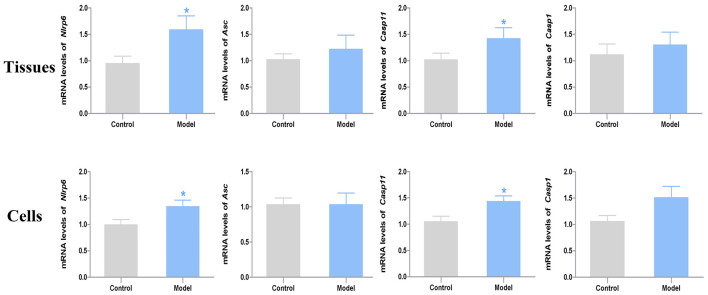
The mRNA expression levels of *Nlrp6, Asc, Casp11* and *Casp1* in the bladder tissues (*n* = 7) and primary nerve cells (*n* = 4). Data represent the means ± SEM (model vs. control group, *P < 0.05).

According to the results of RT-PCR analysis, the mRNA expression levels of *Nlrp6* and *Casp11* were significantly increased in the model group compared with the control group (*P* < 0.05, Cohen’s *d* > 0.8). Of note, the mRNA expression level of *Nlrp6* and *Casp11* in the acrolein-treated neurons exhibited the same tendency compared with the controls (*P* < 0.05, Cohen’s *d* > 0.8). Moreover, the mRNA expression level of *Asc* and *Casp1* from both tissues and nerve cells showed slight difference between the two groups (*P* > 0.05, Cohen’s *d* > 0.2) (Figure 5).

## Discussion

Hemorrhagic cystitis is a common side effect of CYP treatment, but its molecular mechanism remains unknown. The intraperitoneal injection of CYP on murine models has been widely used to explore the development process and pathogenesis of CYP-induced cystitis (Bon et al., 2003; Mickle et al., 2019). Previous studies showed that inflammation in bladder can affect storage and voiding of urine through direct effects on the DSM, influencing innervation to the bladder, altering blood flow, or disrupting the urothelium (Hughes et al., 2014, 2016; Purves and Hughes, 2016; Tudrej et al., 2019). Recently, researchers observed that the gastrointestinal symptoms induced by infection, including reduced motility and loss of intestinal neurons, were mediated by a NLRP6- and CASP11*-*dependent mechanism (Matheis et al., 2020). In the present study, we specifically focused on whether low-dose CYP treatment would jeopardize the anatomic integrity of bladder nerves and alter the expression level of NLRP6 inflammasome in the bladder of rats by *in vivo* and *in vitro* studies.

We measured a series of indicators related to the evaluation of cystitis model. The results of our research showed that, after CYP injections, the model group were characterized by a range of abnormal symptoms, including decreasing body weights, increasing bladder weights and a low level of mechanical withdrawal threshold, which is the same as reported in the literature (Wu et al., 2018; Wang et al). The morphometric analysis suggested bladder inflammation and neuroinflammation in the bladder of the cystitis rat model, confirming our previous conjecture of nerve damage. Urodynamic test revealed that, AMP, PB, peak and PT of model rats significantly increased after CYP treatment. The muscle strips of model rats exhibited significantly higher contractility caused by EFS and CCh than the controls. These results indicated that the model mice entered the stage of overactive bladder (OAB) (Chen et al., 2019; Mills et al., 2020).

Low-dose CYP-induced cystitis model, which exhibited hyperactive urodynamic alterations and enhanced responses of detrusor to EFS and carbachol, have been proved to be a stable OAB model (Juszczak et al., 2009; Mills et al., 2020). Moreover, according to the previous researches in the *in vitro* muscle contractility studies, with short-duration pulses of 50 V and less, the hyper-responsiveness of EFS-induced muscle strip contractions was nerve-mediated and reflected the disordered nerve conduction in the bladder of the model group (McCarthy et al., 2019). Importantly, we found histopathological signs of neuroinflammation in the result of morphometric analysis by transmission electron microscopy. Combined with the results of muscle contractility study, the inflammation in nerve cells may be a major cause of the urinary bladder dysfunction. Other investigators also found denervation in overactive detrusor in clinical and in rodents, and it is believed to be a general feature of pathological fascicles in the overactive bladder (Drake et al., 2003; Kuijpers et al., 2014; Lutolf et al., 2018).

On the other hand, denervation resulting from inflammation might contribute to bladder overactivity. Muscle bundle innervation was derived by dichotomous branching from peri-bundle nerve trunks in the inter-bundle connective tissue. When the denervation is mild, the patchy loss of efferent inhibition would result in unregulated detrusor micromotility. When the denervation is severe, the bladder nervous system becomes largely disconnected with the central nervous system. In conclusion, relatively slight denervation induces detrusor overactivity whereas more severe denervation leads to detrusor underactivity and decompensation (Drake et al., 2017; Lutolf et al., 2018). These may explain the overactive bladder observed in the model group under low-dose CYP treatment.

We noticed that the responses of bladder strips to carbachol, which directly stimulates muscarinic M3 receptors on detrusor muscle, also increased in the CYP treated animals. Considering that the CYP model had significant protein overexpression in detrusor M2 and M3 receptors (Lee et al., 2016). We speculate that there is a compensatory state for overexpressing M2 and M3 receptors on detrusor muscle when the denervation is mild. However, CYP has been well-investigated for damage to the urothelium, which could also partially change activities of bladder detrusor muscle (Andersson et al., 2008, 2012). There are possibilities that altered release of mediators from urothelial cells contribute to the changes in bladder detrusor activities.

The histology test results, including epithelial defect, edema, vascular congestion, and hemorrhage, represent the successful establishment of the CYP-induced cystitis model (Auge et al., 2013; Wang et al., 2017). We have also noticed the abnormal ratio of smooth muscle/collagen. Although the role of connective tissue in bladder function has not been clarified in detail, many previous results suggest that urinary bladder dysfunction is frequently accompanied by abnormal ratio of smooth muscle/collagen (Mirone et al., 2004; Cayan et al., 2008). Therefore, we think this probably is a sign of bladder dysfunction.

EM results show morphological changes of nerves in CYP treated bladder. There isn’t any direct contact between urine and nerve cells in bladder. Moreover, the levels of acrolein formed by metabolism are difficult to quantify in the *in vivo* (or clinical) setting, and may reach very high levels in certain cellular microenvironments, making determination of acrolein challenging (Moghe et al., 2015). Hence, we would like to incubate neurons with acrolein *in vitro* to evaluate the situation of cell apoptosis. However, it is limited to culture pure neurons isolated from bladder, and the major pelvic ganglia are the primary source of postganglionic sympathetic and parasympathetic neurons innervating pelvic organs of rodents. Therefore, we cultured neurons isolated from major pelvic ganglia instead of bladder and incubated them with acrolein. Moreover, we have selected a range of concentration (10–50 μM) previously. However, the apoptotic cells didn’t appear in any of them according to the results of apoptosis assay. Hence, we choose present concentration range. *In vitro* studies showed that, apoptotic cells appeared at the highest concentration group (100 μM acrolein) after 6 h of acrolein incubation in apoptosis assay of primary neurons.

A previous study reported that the NLRP3 inflammasome mediates denervation during bladder outlet obstruction in rats. The study also used *in vitro* analysis to demonstrate that IL-1β, a product of the inflammasome, induces apoptosis in pelvic ganglion neurons (Lutolf et al., 2018). While the best-studied member of the Nod-like receptors (NLRs, one type of the pattern recognition receptors) family is *Nlrp3*, other inflammasomes may have potential roles in bladder inflammatory pathologies as well, through unexplored mechanisms (Guo et al., 2015; Hamilton et al., 2017; Inouye et al., 2018). The NLRP6 inflammasome differs from NLRP3 by not only activating proinflammatory cytokines, but by also having the potential to directly downregulate the NF-κB and mitogen-activated protein kinase pathways (Elinav et al., 2011). Recently, researchers observed long-term gastrointestinal symptoms in murine models of enteric infections, including reduced motility and loss of intrinsic enteric-associated neurons, which were mediated by an NLRP6- and CASP11-dependent mechanism (Matheis et al., 2020). In our study, we found higher mRNA expression levels of *Nlrp6* and *Casp11* in both tissue and primary nerve cells of the model group. This finding suggests that their up-regulation may contribute to the neuroinflammation in the bladder of the CYP-induced cystitis model.

This is a preliminary study to explore the neurotoxicity of low-dose CYP on the urinary bladder of rats. There are still many limitations in this study, such as we used female rats only in this study to avoid functional damage from open surgery in the urodynamic test, and we could not exclude the possibilities that altered release of mediators from urothelial cells contribute to the changes in bladder detrusor activities. Meanwhile, sex hormones might also influence murine and humans when neuroinflammation occurs (Massa et al., 2017). Furthermore, considering the complicated pathogenesis of CYP-induced cystitis, there are many derivative researches worth exploring, such like contribution of various neurotransmitters and relevance between central lesion and neuroinflammation in urinary bladder.

## Conclusion

Our study revealed that low-dose CYP treatment induced nerve injury in the bladder, which leading to pain in the lower abdomen and overactive bladder in female rats, and the up-regulation of mRNA expression levels of *Nlrp6* and *Casp11* may promote these pathological changes.

## Data Availability Statement

The original contributions presented in the study are included in the article/Supplementary Material, further inquiries can be directed to the corresponding author/s.

## Ethics Statement

The animal study was reviewed and approved by the Ethics Committee of Guangzhou University of Chinese Medicine.

## Author Contributions

RW performed the experiments, data analysis, and drafting the manuscript. MH and JH performed the data analysis, methodology, and software. NZ, YZ, JL, JY, LZ, and LH performed the software and data analysis. SX revised the manuscript. BT directed the experiments and revised the manuscript. PH constructed the animal model. H-yC conceived and designed the study. All authors read and approved the final manuscript.

## Conflict of Interest

The authors declare that the research was conducted in the absence of any commercial or financial relationships that could be construed as a potential conflict of interest.

## Publisher’s Note

All claims expressed in this article are solely those of the authors and do not necessarily represent those of their affiliated organizations, or those of the publisher, the editors and the reviewers. Any product that may be evaluated in this article, or claim that may be made by its manufacturer, is not guaranteed or endorsed by the publisher.

## References

[B1] Andersson M. Aronsson P. Doufish D. Lampert A. Tobin G. (2012). Muscarinic receptor subtypes involved in urothelium-derived relaxatory effects in the inflamed rat urinary bladder. *Auton. Neurosci.* 170 5–11. 10.1016/j.autneu.2012.06.004 22789737

[B2] Andersson M. C. Tobin G. Giglio D. (2008). Cholinergic nitric oxide release from the urinary bladder mucosa in cyclophosphamide-induced cystitis of the anaesthetized rat. *Br. J. Pharmacol.* 153 1438–1444. 10.1038/bjp.2008.6 18246091 PMC2437908

[B3] Auge C. Chene G. Dubourdeau M. Desoubzdanne D. Corman B. Palea S. (2013). Relevance of the cyclophosphamide-induced cystitis model for pharmacological studies targeting inflammation and pain of the bladder. *Eur. J. Pharmacol.* 707 32–40. 10.1016/j.ejphar.2013.03.008 23541724

[B4] Bon K. Lichtensteiger C. A. Wilson S. G. Mogil J. (2003). Characterization of cyclophosphamide cystitis, a model of visceral and referred pain, in the mouse: species and strain differences. *J. Urol.* 170, 1008–1012. 10.1097/01.ju.0000079766.49550.94 12913760

[B5] Cayan F. Tek M. Balli E. Oztuna S. Karazindiyanoğlu S. Cayan S. (2008). The effect of testosterone alone and testosterone + estradiol therapy on bladder functions and smooth muscle/collagen content in surgically menopause induced rats. *Maturitas* 60 248–252. 10.1016/j.maturitas.2008.07.008 18774243

[B6] Chen J. L. Zhou X. Ding H. L. Zhan H. L. Yang F. Li W. B. (2019). Neuregulin-1-ErbB signaling promotes microglia activation contributing to mechanical allodynia of cyclophosphamide-induced cystitis. *Neurourol. Urodyn.* 38 1250–1260. 10.1002/nau.24005 30989724

[B7] Drake M. J. Gardner B. P. Brading A. F. (2003). Innervation of the detrusor muscle bundle in neurogenic detrusor overactivity. *BJU Int.* 91 702–710. 10.1046/j.1464-410x.2003.04186.x 12699489

[B8] Drake M. J. Kanai A. Bijos D. A. Ikeda Y. Zabbarova I. Vahabi B. (2017). The potential role of unregulated autonomous bladder micromotions in urinary storage and voiding dysfunction; overactive bladder and detrusor underactivity. *BJU Int.* 119 22–29. 10.1111/bju.13598 27444952 PMC5177525

[B9] Dugani S. Wasan K. M. Kissoon N. (2018). World health organization and essential medicines. *J. Pharm. Sci.* 107 1261–1262. 10.1016/j.xphs.2017.12.019 29277641

[B10] Elinav E. Strowig T. Kau A. L. Henao-Mejia J. Thaiss C. A. Booth C. J. (2011). NLRP6 inflammasome regulates colonic microbial ecology and risk for colitis. *Cell* 145 745–757. 10.1016/j.cell.2011.04.022 21565393 PMC3140910

[B11] Emadi A. Jones R. J. Brodsky R. A. (2009). Cyclophosphamide and cancer: golden anniversary. *Nat. Rev. Clin. Oncol.* 6 638–647. 10.1038/nrclinonc.2009.146 19786984

[B12] Fukuoka M. Negoro S. Masuda N. Furuse K. Kawahara M. Kodama N. (1991). Placebo-controlled double-blind comparative study on the preventive efficacy of mesna against ifosfamide-induced urinary disorders. *J. Cancer Res. Clin. Oncol.* 117 473–478. 10.1007/BF01612769 1909700 PMC12201393

[B13] Guo H. Callaway J. B. Ting J. P. (2015). Inflammasomes: mechanism of action, role in disease, and therapeutics. *Nat. Med.* 21 677–687. 10.1038/nm.3893 26121197 PMC4519035

[B14] Hamann K. Shi R. (2009). Acrolein scavenging: a potential novel mechanism of attenuating oxidative stress following spinal cord injury. *J. Neurochem.* 111 1348–1356. 10.1111/j.1471-4159.2009.06395.x 19780896

[B15] Hamilton C. Tan L. Miethke T. Anand P. K. (2017). Immunity to uropathogens: the emerging roles of inflammasomes. *Nat. Rev. Urol.* 14 284–295. 10.1038/nrurol.2017.25 28266511

[B16] Hara H. Seregin S. S. Yang D. Fukase K. Chamaillard M. Alnemri E. S. (2018). The NLRP6 inflammasome recognizes lipoteichoic acid and regulates gram-positive pathogen infection. *Cell* 175 165–1614.e14. 10.1016/j.cell.2018.09.047 30392956 PMC6294477

[B17] Hughes F. M. Jr. Hill H. M. Wood C. M. Edmondson A. T. Dumas A. (2016). The NLRP3 inflammasome mediates inflammation produced by bladder outlet obstruction. *J. Urol.* 195 1598–1605. 10.1016/j.juro.2015.12.068 26707508 PMC4870136

[B18] Hughes F. M. Jr. Vivar N. P. Kennis J. G. Pratt-Thomas J. D. Lowe D. W. (2014). Inflammasomes are important mediators of cyclophosphamide-induced bladder inflammation. *Am. J. Physiol. Renal Physiol.* 306 F299–F308. 10.1152/ajprenal.00297.2013 24285499 PMC4073918

[B19] Inouye B. M. Hughes F. M. Jr. Sexton S. J. Purves J. T. (2018). The emerging role of inflammasomes as central mediators in inflammatory bladder pathology. *Curr. Urol.* 11 57–72. 10.1159/000447196 29593464 PMC5836190

[B20] Iqubal A. Sharma S. Najmi A. K. Syed M. A. Ali J. Alam M. M. (2019). Nerolidol ameliorates cyclophosphamide-induced oxidative stress, neuroinflammation and cognitive dysfunction: plausible role of Nrf2 and NF- kappaB. *Life Sci.* 236:116867. 10.1016/j.lfs.2019.116867 31520598

[B21] Juszczak K. Ziomber A. Wyczolkowski M. Thor P. J. (2009). Urodynamic effects of the bladder C-fiber afferent activity modulation in chronic model of overactive bladder in rats. *J. Physiol. Pharmacol.* 60 85–91. 20065501

[B22] Kuijpers K. A. Heesakkers J. P. Schalken J. A. (2014). Alterations of the myovesical plexus of the human overactive detrusor. *Biomed. Res. Int.* 2014:754596. 10.1155/2014/754596 24829917 PMC4009145

[B23] Lee W. C. Wu C. C. Chuang Y. C. Tain Y. L. Chiang P. H. (2016). Ba-Wei-Die-Huang-Wan (Hachimi-jio-gan) can ameliorate cyclophosphamide-induced ongoing bladder overactivity and acidic adenosine triphosphate solution-induced hyperactivity on rats prestimulated bladder. *J. Ethnopharmacol.* 184 1–9. 10.1016/j.jep.2015.12.026 26719284

[B24] Lutolf R. Hughes F. M. Jr. Inouye B. M. Jin H. McMains J. C. (2018). NLRP3/IL-1beta mediates denervation during bladder outlet obstruction in rats. *Neurourol. Urodyn.* 37 952–959. 10.1002/nau.23419 28984997 PMC5889355

[B25] Massa M. G. David C. Jorg S. Berg J. Gisevius B. Hirschberg S. (2017). Testosterone differentially affects t cells and neurons in murine and human models of neuroinflammation and neurodegeneration. *Am. J. Pathol.* 187 1613–1622. 10.1016/j.ajpath.2017.03.006 28634006

[B26] Matheis F. Muller P. A. Graves C. L. Gabanyi I. Kerner Z. J. Costa-Borges D. (2020). Adrenergic signaling in muscularis macrophages limits infection-induced neuronal loss. *Cell* 180 64.e–78.e. 10.1016/j.cell.2019.12.002 31923400 PMC7271821

[B27] McCarthy C. J. Ikeda Y. Skennerton D. Chakrabarty B. Kanai A. J. Jabr R. I. (2019). Characterisation of nerve-mediated ATP release from bladder detrusor muscle and its pathological implications. *Br. J. Pharmacol.* 176 4720–4730. 10.1111/bph.14840 31430833 PMC6965683

[B28] Mickle A. D. Won S. M. Noh K. N. Yoon J. Meacham K. W. Xue Y. (2019). A wireless closed-loop system for optogenetic peripheral neuromodulation. *Nature* 565, 361–365. 10.1038/s41586-018-0823-6 30602791 PMC6336505

[B29] Mills K. A. West E. J. Grundy L. McDermott C. Sellers D. J. Rose’Myer R. B. (2020). Hypersensitivity of bladder low threshold, wide dynamic range, afferent fibres following treatment with the chemotherapeutic drugs cyclophosphamide and ifosfamide. *Arch. Toxicol.* 94 2785–2797. 10.1007/s00204-020-02773-2778 32444959

[B30] Mirone V. Imbimbo C. Sessa G. Palmieri A. Longo N. Granata A. M. (2004). Correlation between detrusor collagen content and urinary symptoms in patients with prostatic obstruction. *J. Urol.* 172(4 Pt 1), 1386–1389. 10.1097/01.ju.0000139986.08972.e3 15371851

[B31] Moghe A. Ghare S. Lamoreau B. Mohammad M. Barve S. McClain C. (2015). Molecular mechanisms of acrolein toxicity: relevance to human disease. *Toxicol. Sci.* 143 242–255. 10.1093/toxsci/kfu233 25628402 PMC4306719

[B32] Purves J. T. Hughes F. M. Jr. (2016). Inflammasomes in the urinary tract: a disease-based review. *Am. J. Physiol. Renal Physiol.* 311 F653–F662. 10.1152/ajprenal.00607.2015 27170685 PMC5142237

[B33] Rzeski W. Pruskil S. Macke A. Felderhoff-Mueser U. Reiher A. K. Hoerster F. (2004). Anticancer agents are potent neurotoxins in vitro and in vivo. *Ann. Neurol.* 56 351–360. 10.1002/ana.20185 15349862

[B34] Tudrej K. B. Piecha T. Kozlowska-Wojciechowska M. (2019). Role of NLRP3 inflammasome in the development of bladder pain syndrome interstitial cystitis. *Ther. Adv. Urol.* 11:1756287218818030. 10.1177/1756287218818030 30671141 PMC6329030

[B35] Wang H. J. Lee W. C. Tyagi P. Huang C. C. Chuang Y. C. (2017). Effects of low energy shock wave therapy on inflammatory moleculars, bladder pain, and bladder function in a rat cystitis model. *Neurourol. Urodyn.* 36 1440–1447. 10.1002/nau.23141 28035695

[B36] Wu K. C. Chiang B. J. Tsai W. H. Chung S. D. Chien C. T. (2018). I-Tiao-Gung extract through its active component daidzin improves cyclophosphamide-induced bladder dysfunction in rat model. *Neurourol. Urodyn.* 37 2560–2570. 10.1002/nau.23815 30252154

