# Supplementary material for: Low-Dose Cyclophosphamide Induces Nerve Injury and Functional Overactivity in the Urinary Bladder of Rats
Source: Front Neurosci. 2021 Oct 1;15:715492. doi: 10.3389/fnins.2021.715492 (PMC8517437; doi:10.3389/fnins.2021.715492)
Supplement: Supplementary file 1 [file Table_1.docx]

Fig 1 raw data and original images

<https://www.jianguoyun.com/p/DSwonvgQx_nFCRjQ_Y0E>

<https://www.jianguoyun.com/p/DY8sdm0Qx_nFCRjJ4vcD>

Fig 2 raw data and original images

<https://www.jianguoyun.com/p/DQfaQOUQyfnFCRjU4vcD>

<https://www.jianguoyun.com/p/DQR0ezQQyfnFCRjc4vcD>

Fig 3 and raw data

<https://www.jianguoyun.com/p/DYTwI3YQ9OPpCRj6u48E>

<https://www.jianguoyun.com/p/DQzFzXsQ9OPpCRj8u48E>

Fig 4 immunofluorescence images

<https://www.jianguoyun.com/p/DdGaLLMQzvnFCRi-3_cD>

<https://www.jianguoyun.com/p/DT55xzcQzvnFCRi_3_cD>

Fig 5 and raw data

<https://www.jianguoyun.com/p/DSTCXSAQ0fnFCRjO940E>
